# 

**Published:** 1818-12

**Authors:** 


### BOOKS PREPARING FOR PUBLICATION.

Practical Observations on the Construction and Principles of Instruments for the Removal of Muscular Contraction of the Limbs, Distortion of the Spine, and every other species of Personal Deformity, by JOHN FELTON, (late of Hinckley,) 1 vol. 8vo.

Mr. COAR's edition of the Aphorisms of Hippocrates, in Greek, Latin, and English.

A Treatise on Midwifery, enforcing new principles, which tend materially to lessen the sufferings of the patient, and shorten the duration of labour, by JOHN POWER, accoucheur.

Mr. PARKINSON's Familiar Introduction to the Study of Fossils.

Synopsis Zoo-Nosologia, or the Elements of Medical Science (an entirely new system), with an appropriate Nomenclature, by Dr. PARKINSON.

The first part of a work, by Dr. WATT, of Glasgow, entitled Bibliotheca Britannica, or a general Index to the Literature of Great Britain and Ireland, ancient and modern, with such foreign works as have been translated into English or printed in the British dominions; also a copious selection from the writings of the most celebrated authors of all ages and nations.

Cases, with Observations on Wry-neck, on the Reduction of Luxation of the Shoulder-Joint, on the Operation for Hare-lip, on Cartilaginous Substances of the Knee-Joint, on Aneurism, and on the use of the Extract of Stramonium, by JOHN KIRBY, A.B. &c. in 8vo.

Observations on Inflammation of the Mucous Membrane of the Respiratory Organs, illustrative of the pathology and treatment of Bronchial Inflammation, Croup, Hooping-cough, Measles, Catarrh, and those affections resembling Pulmonary Consumption, &c. exemplified by cases, dissections, and coloured engravings; by THOMAS ALCOCK, surgeon.

No. I. of a New System of Medical Botany, handsomely printed in royal 8vo. with plates coloured according to nature, and botanical descriptions of each plant, giving the place of its growth, medical properties, dose, &c.

---

### *NOTICES TO CORRESPONDENTS.*

*Several Communications have been received, which our limits would not permit us to insert in the present Number, and we trust the authors of them will not feel offended at their being deferred until the second of the ensuing Volume; the length of the Historical Sketch of the general Progress of Medical Knowledge during the last half-year, with which the first Number will commence, will render such an arrangement absolutely necessary.*

*Although we have given an additional Half sheet in the present Number, we have been obliged to transfer our* LISTS OF BOOKS *and* LECTURES *to the* COVER, *for want of room.*

*We feel it requisite to inform our readers, that Memoirs on subjects respecting which it is desirable that information should be immediately made public, should be transmitted to us before the 12th of the month, in order that they may appear in the ensuing Number. We, however, owe an apology to* Dr. BENT *for having deferred the insertion of his paper until the present time; although it was received about the period to which we have alluded, yet the time required for the execution of the coloured Engraving, prevented its appearance in our last Number.*

---

ERRATA.

Page 102, line 15, for *atonic* read *atomic.*
— 259, — 1, dele *Medical and Surgical.*
— 266, — 32, for *the cure was evident,* read *the case,* &c.
— 273, — 46, for *inches square,* read *square inches.*
— 302, — 28, *president,* add, *of a society composed of the members of.*
— 322, — 35, for *W.* read *A. C.*
— 339, — 35, after 30° add *Reaumur,* or read 100° *Fah.*

### INDEX

### TO THE FORTIETH VOLUME.

### ANATOMY.

Page

RETINA, observations on the structure of it, by Mr. Littleton 178
PELVIS, Mr. C. Bell's remarks on the axis of the 223
An instance of unusual distribution of the thoracic and abdominal viscera 339

### PATHOLOGY, AND PATHOLOGICAL ANATOMY.

STRICTURE OF THE COLON, with rupture of that intestine 15
INSANITY, the opinions of Dr. Parkman respecting its analogy to sanity 121
———, various disordered impulses of the will in ib.
———, disposition to suicide in 122
———, remarkable change of countenance in, previously to its appearance 124
———, sensibility and insensibility to heat and cold in ib.
MUSCÆ VOLITANTES, remarks on, by Mr. Littleton 182
NOSOLOGY, Dr. Parkinson's strictures on 183
SPASM, observations on its influence in the production of disease, by Dr. Wilson 247
IRRITATION, produces many effects through the medium of nervous sympathy 249
TETANUS, induced by disease of the spinal marrow 259, 303
CALCULUS, case of, in a bitch, by Dr. Maclean 288
STOMACH, ulceration and rupture of 295
———, rupture of, from the action of vomiting 532
TUMORS, morbid 62, 297, 300

Page

LIVER, DISEASED, from habitual drunkenness 299
LIVER, abscess in 306
INFLAMMATION OF THE BRAIN, species of, as defined by Dr. Abercrombie 315
CYNANCHE LARYNGEA, pathological observations on, by Dr. Bliss 335
GRAVEL, causes of the production of 339
PATHOLOGICAL OBSERVATIONS, by Dr. Lallemand 343
EXTRA-UTERINE GRAVIDITY, case of ib.
PLACENTA, facts tending to elucidate the mode of its development 344
PARALYSIS, arising from solution of continuity of the brain 351
BRONCHOCELE, question respecting the existence of, in the fœtus 352
CHANGE OF COLOUR OF THE SKIN in an American Indian 391
———, a similar case related by Dr. Rush 392
HEART, rupture of 425
———, ANEURISM OF, observations on Corvisart's divisions of that disease into active and passive 443
———————, pathological facts respecting 444
HÆMORRHAGE, family predisposition to, instances of, related by Drs. W. and S. Buel 429
ACEPHALI, the opinion of Morgagni respecting the cause of the destruction of the brain in the fœtus;—that it ensues from hydrocephalus 431
———, instances of 346, 434
——— ; instance of one that lived four days from the time of its birth 439
———————, anatomical description 346

SPINAL MARROW, the opinions on the causes of its destruction in the fœtus, of Morgagni, Lallemand, and Brunner 432
————, *cavity formed in*, opinions respecting it, of Malpighi, Portal, and Gall 432
————, conclusion of M. Lallemand respecting it ib.
VARIOLA AND VARICELLA; facts tending to shew their identity 457
REFLECTIONS on the medical measures instinctively employed by brute animals 486
FACTS tending to prove the influence of sympathy between the genitals and the throat and skin, in the production of disease 507
AORTA, instance of obliteration of its canal 515
VEINS, effects of wounds and ligatures of them 521
————, the mode in which the union of wounds of veins takes place 523
————, the effects of ligatures ib.
————, causes of inflammation of veins 524
————, terminations of inflammation of veins 525
PULSATION IN EPIGASTRIO; the various causes productive of it 529
DIGESTION; circumstances respecting that process in persons affected with preternatural anus 533
DISUNION OF A RECENTLY-UNITED FRACTURE OF THE OS HUMERI 538
DISPLACEMENT OF THE COLON, observed by M. Esquirol in cases of insanity 542
ATTEMPT TO SHOW THE IDENTITY OF SIBBENS, and the disease that appeared in Europe in the year 1496, and generally considered as syphilis 546

### PHYSIOLOGY.

RESPIRATION, observations of Van Swieten's description of, by Mr. Littleton 31
————, case showing that the action of the muscles of one side may be suspended in this act, while those of the other side perform their functions 291
PULSE, observations on the, by Mr. Littleton 32
SANITY, the opinion of Dr. Parkinson respecting its relation to insanity 121
NERVES; cases in which those of the arm were included in ligatures after amputation, with the consequences, related by Mr. Hennen 215
NERVOUS SYSTEM, observations on the functions of the, by M. Lallemand 346
————, comparison of that of man with that of other animals 440
NERVOUS POWER; the opinions of Reil, Prochaska, Scarpa, and Legallois, respecting its being produced in the nerves themselves 438
NERVOUS INFLUENCE of the system of animal life, axioms respecting 44[illegible]
SYMPATHETIC NERVE; facts tending to prove, that that nerve is alone necessary for the functions of organic life 346
VISION, Mr. Littleton's remarks on 177
————, remarks on the opinions respecting the muscular power of the fibres of the crystalline lens ib.
————, observations on the structure and functions of the retina 179
————, observations on the refractive powers of the different parts of the eye, by Mr. Littleton ib.
INEDIA, observations on, by Mr. Good 289
DECARBONISING FUNCTION of the lungs and other organs, observations on, by Dr. Pierson 331
URINE, the influence of azotid aliments on the qualities of 340
DECIDUARY MEMBRANE found in the uterus, in cases of extra-uterine gestation, by Chaussier, Meckel, and Weinknecht 344
MECONIUM; the opinion of M. Lallemand respecting its production 233
INGURGITATION, observations on 393
LIFE, in the fœtus, wholly employed in the development of its organs 435
————, Mr. Brodie's experiments to ascertain the mode in which the vegetable poisons destroy it 436
————, facts seeming to prove that the brain is neither the sole source of nervous influence, nor the sole centre of the nervous system of animal life 439
LIFE, independent of the influence of the brain and spinal marrow, in the fœtus 435
——, ANIMAL, interesting facts respecting, by M. Lallemand 439
IRRITABILITY; observations on the doctrine of Haller 434
HEART; enquiry respecting the origin of its nervous influence, and remarks of Legallois respecting it 437
ACTION OF THE VOLUNTARY MUSCLES of a fœtus without brain or spinal marrow 438
SYMPATHY, the immediate cause of death in cases of severe injury of the brain 440
TEMPERATURE, change of, in the boa constrictor serpent 451
PROTEUS ANGUINUS, observations respecting 452
DIGESTION; elective action of the stomach 531
————; facts showing the elective action of the stomach in this process 534
————; facts showing that substances containing the most nutriment are longest retained in the stomach, and most perfectly altered before their escape from that organ 535
————, the action of the intestines in 537

MEDICINE.

TRISMUS, case of, accompanied with hysteria, by Dr. Maclean 15
MENSES, suppressed, the beneficial effects of black hellebore in 16
HYSTERIA, cases of, from diseased liver, by Dr. B. 23
PERTUSSIS, observations on, by Mr. Littleton 26
————, observations relative to the origin of 28
————, on the favourable and unfavourable symptoms of 30
————, recurrence of, in the same person 31
————, opinion of Mr. L. on the necessity of actual contact for the communication of this disease ib.
PHTHISIS, mode of prevention of, by Mr. Lauthois 73
INSANITY, remarks on, by Dr. Parkman 120
————; abstract of the doctrine of Pinel 479
————; analogy between sanity and insanity 121
————; observations on excessive blood-letting in maniacal affections, by Dr. Kinglake 368
————, on the causes of 123
————; dread as a cause ib.
————; intemperance ib.
————; religious enthusiasm 126
————; undue activity of the imagination ib.
————; grief consequent on being separated from friends, &c. ib.
————; anxiety of mind ib.
————; various other mental emotions ib.
————; observations of Dr. Parkman on the means of ascertaining the existence of it 188
————, Dr. P.'s remarks on the use of restraint in ib.
————, M. Pinel's ditto 397
————; on restraint, as employed at the asylum at Avignon 298
————; Dr. Parkman's remarks on periodical exacerbations of it 189
————; on appropriate places of residence for lunatics 190
————, medical treatment of ib.
————, the importance of attending to the spontaneous salutary changes of the system in 191
————, use of mercury in 192
————, M. Esquirol's observations on 543
INFLUENZA; notice respecting the discussion of the subject in this Journal 129
GOUT, remarks on Dr. C. Wilson's Specific for, by Dr. Williams 146
SYMPATHIES, MORBID, Dr. Andrew Wilson's practical observations on 242
————————, account for many phenomena observed in diseases 244
————————, dependent on the influence of the brain and nervous system 246
————————, forms and appearances of 249
————————, frequently constitute the principal symptoms of disease 405

TRANSACTIONS OF THE ASSOCIATION OF THE FELLOWS AND LICENTIATES OF THE COLLEGE OF PHYSICIANS OF DUBLIN 294
LIVER, cases of suppuration of 298
ANGINA PECTORIS, case of, relieved by the inhalation of oxygen gas, by Dr. Reid 303
MELÆNA, cases of, by Dr. Brooke 304
DROPSY, accompanied with inflammation of the lungs, &c. observations by Dr. Stoker 305
———, *ovarian*, case of, by Dr. M'Loghlin 306
RHEUMATISM, *chronic*, case of, cured by bandages, by Dr. Grattan 305
———, *intermittent* 322
TRANSACTIONS of the Physico-Medical Society of New-York 331
CYNANCHE LARYNGEA, observations on, by Dr. James C. Bliss 335
FEVER, Dr. Wilson's opinions respecting its nature 406
———, case of, tending, in the opinion of Mr. Scott, to support the doctrine of critical days 279
———, observations on, by Dr. Percival 307
———, unfavourable symptoms of 312
———, on the crisis of ib.
———, diagnostic signs from the appearance of the tongue in ib.
———, treatment of 313
———, Dr. O'Brien's remarks on 398
———, Dr. Grattan's 399
———, instance of the sudden supervention of inflammatory symptoms to the mild form of, at an advanced period of the disease 498
———, *typhous*, on the prevention of, by Dr. Fergusson 21
———, accompanied with disease of the spinal marrow 321
———, remarks on the pathology and treatment of, by Dr. Callanan 491
———, case illustrative of the simple, making a sudden transition into one of the more complex, forms 496
———, instance of congestive 499
———, its division into *simple*, *inflammatory*, and *congestive* 493
———, history of the symptoms of, and its terminations 500
FEVER, *hydrocephalic*, case of, by Dr. Crampton 306
———, *epidemic*, of Dublin
———, division of, into genera, considered improper 311
———, Dr. Grattan's classification of the species of 401
———, *vernal*, character of 308
———, *summer* ib.
———, *autumnal* 309
———, *winter* ib.
———, *yellow*, opinions respecting the contagious nature of, by MM. Hallé, Leroux, and Chaussier 82
———, on the temperature of the summers which give rise to, in Philadelphia 205
BRAIN, case of disease, by Dr. Johnson 447
———, *inflammation of the*, case of, by Mr. Parkinson 474
———, cases of, with observations, by Dr. Abercrombie 315
———, divided into four species 316
SPASMODIC DISEASES, on the use of emetics in their treatment, by Dr. Smith 336
GRAVEL, researches respecting, by Dr. Majendie 338
———, causes of 339
———, effect of azoted diet in the production of 340
———, curative indications 341
PARALYSIS, cases of, by Dr. Serres 351
AMENORRHŒA, observations on 381
PHLEGMASIA DOLENS, case of, by Dr. Purdy 426
NYMPHOMANIA, case of, by Dr. Jauzion 441
HEART, *aneurism of*, cases, with observations by M. Rostan
———, abscess and rupture of, by Dr. Mott 428
VARIOLOID DISEASE; observations on the varioloid disease prevalent in the county of Derby, in the year 1818, by Dr. Bent 458
HEPATIC CONGESTION, observations on, by Dr. Kinglake 463
TIC DOULOUREUX, observations on, by Dr. Leslie 476
SCARLATINA, observations on, by Dr. Parkinson 477
HYDROCEPHALUS, on the good effects of moxa in its treatment, by Dr. Regnault 529

HYDROCEPHALUS, case of, with observations by Dr. Brooke 301
MONTHLY REPORTS OF DISEASES, 85, 176, 261, 357, 455, 550

### CHIRURGERY.

DUBLIN HOSPITAL REPORTS 62
PERIOSTITIS, Essay on, by Mr. Crampton 64
———, different forms of ib.
———, terminations of 65
———, origin of ib.
———, case of the acute form, from external injury ib.
———, terminating fatally, by extension of the disease to the dura mater 66
———, case terminating in thickening of the periosteum 69
———, cases of the sub-acute form ib.
——— chronic form 71, 72
URETHRA, on diseases of, by Mr. C. Bell 139
——— and BLADDER, affections of, arising from disorder of the alimentary canal, by Mr. Bell 145
SURGERY, MILITARY, Mr. Hennen's observations on 154
———, historical sketch of the progress of ib.
GUN-SHOT WOUNDS, Mr. Hennen's remarks on the nature of 157
——— the treatment of 158, 160
SURGICAL OBSERVATIONS, by Mr. C. Bell 48
AMPUTATION; Mr. Hennen's opinion in favour of the *immediate* performance of the operation, in cases in which it would eventually be necessary 159
———, general and particular remarks on, by Mr. Hennen 221
FOREIGN BODIES, on the introduction of, into the body, from gun-shots 161
CONTUSIONS, Mr. Hennen's remarks on 162
FRACTURES, *compound*, Mr. Hennen's remarks on 169
BONES, injuries of, from gun-shot wounds 162
———, peculiar injury of, from gun-shot, described by Mr. Hennen 163
JOINTS, injuries of, from gun-shots 164
———, extraordinary recovery from a case of severe injury of the knee-joint from gun-shot, related by Mr. Hennen ib.
BLOOD-VESSELS, injuries of, from gun-shot 214
LIGATURE, historical account of, and of the short-cut ligature ib.
———, consequence of including the nerves in, after amputation 215
ANEURISM, *varicose*, case of, from gun-shot wound, related by Mr. Hennen ib.
———, its fatal termination from tying the artery ib.
NERVES, injuries of, from gun-shot wounds; cases, and remarks on by Mr. Hennen ib.
GANGRENE, HOSPITAL, Mr. Hennen's remarks on 216
———, sketch of topography respecting ib.
———, rapid communication and progress of 217
———, appearance of the disease in its incipient stage 218
———, progress of, in the advanced stages ib.
———, case of death of a soldier within twenty-four hours from his admission into the hospital, at which period he was free from the disease 219
———, treatment of ib.
———, the beneficial effects of bleeding in, as employed by Mr. Hennen 219
———, Mr. Hennen's remarks on the use of Fowler's solution of arsenic in the treatment of ib.
———, *see Phagedæna Gangrænosa.*
MORTIFICATION from gun-shot wounds, Mr. Hennen's remarks on 220
———, Larrey's division of ib.
———, Mr. Curtis's observations referred to ib.
TETANUS, observations on, when arising from gun-shot wounds, by Mr. Hennen ib.
———, peculiar smell of the body in, observed by Mr. Hennen ib.
———, cases of, by Dr. Franck 259
———, case, with observations, by Dr. Reid 303

WHITE-SWELLING, Mr. C. Bell's observations on 222
————, treated by setons passed through the joint, by Mr. C. Bell 223
LITHOTOMY, Mr. Bell's remarks on 225
————, the steps of the operation when performed with the knife instead of the gorget ib.
PHAGEDÆNA GANGRÆNOSA, *(Hospital Gangrene,)* Mr. H. Blackadder's observations on 226
————; topography of the situation in which it appeared 226, 227
————; appearance of phagedæna gangrænosa with constitutional derangement 228
————; general mode of treatment formerly adopted by military surgeons ib.
————; Mr. Blackadder's opinions respecting the mode in which it is developed,—that it arises from local contact of a morbid poison 229
————; those of Professor Brugmans, Moreau, J. Bell, and Mr. Hennen 230
————, origin of, from the use of foul lint, as described by Prof. Brugman and Pelletier 231
————; origin from the use of surgical instruments tainted with the matter issuing from ulcers affected with, as noticed by Pouteau ib.
————; Mr. Blackadder's mode of treating it by cauterizing applications ib.
————; the beneficial effects of those means ib.
————, history and description of, by Mr. Blackadder 232
————; researches respecting its history, as it is to be found in the writings of ancient and modern authors 239
AMAUROSIS, *periodical*, case of, cured by cinchona, by Mr. Austin 267
EXCISION OF A PORTION OF THE RIBS AND PLEURA, by M. Richerand 272
BURNS AND SCALDS, Mr. Nodes Dickinson's remarks on 417
————, Mr. D.'s remarks on Dr. Kentish's plan of treatment 420, 424
NASAL OPERATION, case of 322
INSTRUMENTS, *surgical*, an essay to improve the construction of them, by Mr. Jardine 324
UTERUS, case of prolapsus of, in which that organ was extirpated, by Mr. Newnham 325
ELEPHANTIASIS, case of 353
TUMOR of the os humeri, case of, by Mr. Baldy 364
————, removal of, from the angle of the jaw, by Dr. Cusac 62
CANCER, case of, terminating fatally by trismus 386
PRETERNATURAL JOINT, cured by friction 427
————; general account of the results of the use of the seton in that disease 428
SPINAL DISTORTION; observations respecting it 450
SURGICAL ESSAYS, by Mr. Cooper and Mr. Travers 502
On DISLOCATIONS, by Mr. Cooper 504
On IRITIS, by Mr. Travers ib.
————, on the origin of iritis 505
————, on its connexion with syphilis, and the influence of mercury on the system 506, 508
————, appearance of the disease when arising from syphilis 509
————, the mode of treatment employed by Mr. Travers ib.
History of a Case of LIGATURE OF THE AORTA, by Mr. Cooper 511
PHYMOSIS AND PARAPHYMOSIS, observations on, by Mr. Travers 515
EXOSTOSIS, observations on, by Mr. Cooper 516
————; the bones most generally affected with it 517
————; description of the fungous exostosis of the medullary membrane ib.
————; cartilaginous exostosis of the medullary membrane 518
————; fungous exostosis of the periosteum 519
————; cartilaginous exostosis of the periosteum ib.
ANEURISM, cases of carotid and brachial, cured by ligatures, by Dr. Post 531

CALCULUS; mode of extracting it from the bladder through the urethra in the male, by Dr. Conant 545
SYPHILIS, remarks on the antiquity of, by Mr. Beetson 193
———, Mr. Alcock's observations on 170
———, on its cure without mercury 320
———, Mr. Osbeck's mode of treating by spare diet 340
———, question respecting its communication to the fœtus 352
———, mode of treatment employed in the sixteenth century 374, 470
———; Mr. C. Bell's remarks on 48
HERNIA, observations on, by Mr. Todd 151
———; case in which the spermatic cord passed before the hernial sac ib.
———; similar cases referred to, related by Blizard, Schmucker, and Le Dran ib.
———; remarks by Mr. Todd on the species of hernia first described by Mr. Hey, in which the viscus protrudes within the tunica vaginalis of the cord, but covered by another elongation of the peritonæum 151
———; Mr. Todd's objections to Mr. Hey's history of the above disease, and his own opinions respecting it 152
———, observations on the state of the peritonæal sac in, by Mr. C. Bell 224
———, *crural*, remarks on, by Mr. Todd 153
———; observations on Mr. Cooper's and Mr. Lawrence's account of the situation of the protruded viscus with respect to the fascia ib.
———; on the division of Gimbernat's ligament in the operation for ib.
———, *umbilical*, case of, by Dr. Girdlestone 13
———, *congenital*, with a peritoneal sac enveloping the protruded intestine, by Mr. Hunt 265
———, *strangulated*, case of, terminating in sphacelus, and cured without being followed by permanent preternatural anus, by Dr. Duplan 543
OPERATIONS, *surgical*, Mr. Hunter's sentiment respecting 327

### MIDWIFERY.

On the importance of the profession of midwifery, by Mr. Wales 17
Case of death from lingering labour, with the remarks of Mr. Wales 19
LYING-IN HOSPITAL OF DUBLIN, abstract from the registry of, by Dr. Clarke 401
PARTURITION, *laborious*, results from 402
HÆMORRHAGE, *uterine*, cases of, and results of different modes of treatment ib.
———, case of, and observations on, by Mr. Purton 123
CONVULSIONS, *puerperal*, general results of different modes of treatment in 403
LACERATION of the urethra and bladder ib.
——— the vagina and uterus ib.
PROLAPSUS of the funis ib.
PLACENTA; communication between the vessels of the placentas in cases of twins 345
———; on the measures which tend to prevent its retention 401
———, cases of retention of 403

### CHEMISTRY.

RETROSPECT of the progress of Chemistry 89
BLOW-PIPE 91
THERMOMETER 92
GASOMETER ib.
CRYSTALLIZATION of bodies, Dr. Ure's researches into the laws of the ib.
CLASSIFICATION of mineral substances, Mr. Bendant's method 94
MAGNETISM as a means of detecting the presence of iron, Mr. Haüy's experiments on 95
LIGHT, Dr. Brewster's observations on the laws of its polarization in crystallized bodies 96
———, Morrichini's experiments of the communication of magnetism by the violet rays ib.

LIGHT, M. Berard's experiments on the properties of the different rays 96
FLAME, the doctrine of, by Sir Humphrey Davy 97
CHLORINE, Dr. Ure's researches into the nature of 98
———, Dr. Murray's ditto ib.
———, Sir H. Davy's recent ditto 100
HYDROGEN GAS, Dr. Murray's notion respecting its influence in the production of acidity 99
———, its effects on oxide of silver 168
IODINE, mode of preparing it from soap-leys 100
———, crystallized 168
HYDRIODIC ACID, M. Houton Labillardiere's experiments with 100
OXALIC ACID, M. Bauhof's experiments with 101
PHOSPHURETS, Mr. Dalton's experiments with 102
PHOSPHORUS, experiments on its combinations with oxygen and chlorine, by Sir H. Davy ib.
ALKALINITY, Dr. Murray's opinions respecting the influence of oxygen and hydrogen in the production of 103
MANGANESE, reduced to the metallic state by M. Fischer 104
IRON, Dr. Marshall Hall's experiments on the action of water and oxygen on ib.
URANIUM, ascertained to be soluble in the alkaline sub-carbonate, by M. Chevreul 104
COPPER, native mass of ib.
———, experiments on the carbonates of, by Mr. Phillips 105
MERCURY, new mode of preparing its submuriate 106
PLATINA, sulphuret of ib.
———, submuriate of ib.
———, chloride of 107
——— and soda, muriate of ib.
———, sulphate of ib.
ARSENIC; the means of detecting it in minute quantities 108, 281, 407
CHROMIC ACID AND OXIDE, phenomena respecting 108
PARGASITE, a new mineral from Finland 108, 172
LEELITE, ditto, from Sweden 109
PETALITE, ditto, ditto ib.
LITHINA, ditto, ditto 109, 134
SELENIUM, ditto, ditto 110
CADMIUM, a new metal 112, 256
MINERAL WATERS of Caldas da Rainha 11
ESSENTIAL OIL of nut-galls 113
MORPHINE, *the alkaline salt of opium* 114
EMETINE, *the alkaline salt of ipecacuanha* ib.
INDIGOGENE 115
ANIMAL CHEMISTRY 117
FAT, experiments of M. Berard on the formation of 118
BLOOD, on the colouring matter of, by Berzelius ib.
MOIRE METALLIQUE, mode of effecting it 169
LEAD, oxide of, crystallized 168
COAL, new products from, obtained by Dr. Jassmeyer 169
——— gas, use of, for the blowpipe 172
SILICA and ALUMINA, hydrate of, discovered by M. Dufour 169
———, siliciferous sub-sulphate of alumina, described by Dr. Henry ib.
EARTH, RED, shower of, in Italy, with a chemical analysis of it by Sig. Sementini 171
DIAMOND ROCK, Dr. E. D. Clarke's analysis of a specimen of 172
METEORIC STONES, Mr. Brande's observations on 250
LITHIC ACID, physical and chemical properties of 338
CYSTIC OXIDE, physical and chemical properties of 339
PYRO-LIGNEOUS ACID, its antiseptic qualities 354
PRUSSIC ACID; observations on the mode of preparing it 449
HYDROGURETTED CARBONIC OXIDE 453
ALCOHOL, production of, from potato-apples ib.

### MATERIA MEDICA.

MORPHINE; the mode of exhibiting it, and cases to which it is particularly applicable 114, 275
BLACK HELLEBORE, its use in removing suppression of the menses 16
Observations on the use of YEAST and CARBONIC ACID GAS, in typhus, by Dr. Ferguson 22
TARTAR EMETIC, Dr. Balfour's remarks on 410
———, cases illustrative of its good effects 413

Tartar emetic, used by M. Lanthois for the prevention of consumption 73
Alisma plantago; its use in hydrophobia 76
————————, botanical description of 453
Nitrate of silver; cases in which it was employed with advantage by Dr. Balfour ib.
Tar-vapour; its use in asthma 82
Digitalis; its use as a local application to scrofulous ulcers 119
————, its use in epilepsy 204
Blood of a rabid animal employed in Russia to prevent the usual effects of its bite 119
Enemas, remarks on the utility of, by Mr. Machell 198
————, description of a new machine for the administration of 200
Soda, sub-borate of, its use in ulcers 207
Belladonna, on the medicinal qualities of, from the *Dict. des Sciences Med.* 173
Remedy for gout 292
Oxygen gas, its use in angina pectoris 303
Antimonial powder, observations on the medical qualities of 353
Proposal for a new mode of preserving vegetable remedies, by Dr. Marshall Hall 354
Observations on the use of patent medicines 389
Lauro-cerasus, on the medicinal properties of 395
Colchicum, observations on its use in gout, by Sir Everard Home 396
Prussic acid, deleterious power of 450
Actual cautery, observations respecting 451
Pyrola umbellata, observations on the medicinal qualities of ib.
Pectoriloqual instrument of Laennec ib.
Uva ursi, its botanical character and medicinal qualities 468
Oil of turpentine, its use for the destruction of tænia 546
Oil of turpentine, its good effects in cases of melæna 304

### MISCELLANEA.

Case of recovery of a patient after swallowing a large quantity of sulphuric acid, by Mr. Browne 25
Biography of Dr. Woodville 34
———— of Dr. Rush 39
Medical benevolent society, meeting of 74
Associated apothecaries of England and Wales, meetings of 75, 167
Case of recovery after taking a large quantity of arsenic 75
Dwarf, account of a female 81
Monstrous fœtus 84
Necrology of Dr. Adams 85
———— of Dr. Merriman and Dr. Wilkinson 356
Lamp, mode of constructing without flame 98
Case of a child who swallowed a knife, without experiencing permanent serious injury 209
Botanical productions in the neighbourhood of the River Zaire 355
On the population of England 209
On the sounds produced by flame in tubes, by Mr. Faraday 129
Historical account of meteoric stones, by Prof. Brande 137
Sitting of the Royal Society, June 4 163
Account of the Dublin Medical School 253
———— the Imperial Society of Naturalists at Moscow 254
On the colours of bodies, by M. Prevost 256
Mouse, influence of music on 257
On the milk of the Cow-tree, by M. Humboldt 258
Specific gravity, observations on, by Mr. Littleton 269
Water-spouts, observations on, by Captain Lynn 78
Prize questions 549

### NAMES OF AUTHORS,

*Whose Literary Productions are inserted in the Fortieth Volume of the*

**LONDON MEDICAL AND PHYSICAL JOURNAL.**

ABERCROMBIE, Dr. observations on Inflammation of the Brain.

AUSTIN, Mr. case of periodical Amaurosis.

BALDY, Mr. case of Cartilaginous Tumor of the Os Humeri.

BALFOUR, Dr. Observations, with Cases, illustrative of the sedative and febrifuge powers of Emetic Tartar.

BEATSON, Mr. observations on the antiquity of Syphilis.

BELL, Mr. Surgical Observations.

BENT, Dr. observations on the Varioloid Disease prevalent in the county of Derby.

BISSEL, Dr. account of the change of colour of the skin of an American Indian.

BLACK, Dr. case of gouty affection, and account of the dissection of two habitual drunkards.

BLACKADDER, Mr. H. HOME, Observations on Phagædena Gangrænosa.

BLISS, Dr. observations on Cynanche Laryngea.

BRANDE, Prof. observations on meteoric stones.

BROOKE, Dr. cases of Hydrocephalus and Melæna, with observations.

BROWNE, Mr. case of Recovery of a Person after having swallowed a large quantity of Sulphuric Acid.

BUEL, Drs. W. and S. history of a family predisposition to Hæmorrhage.

CALLANAN, Dr. observations on the pathology and treatment of Typhous Fever.

CLARKE, Dr. abstract from the Registry of the Dublin Lying-in Hospital.

CONANT, Dr. new mode of extracting Stone from the Bladder.

COOPER, Mr. Surgical Essays.

CRAMPTON, Dr. histories of a case of Ulceration and Rupture of the Stomach, and of Hydrocephalic Fever.

CUSACK, Dr. account of the Removal of a Tumor from the Neck.

DICKINSON, Mr. remarks on Burns and Scalds.

DUPLAN, Dr. case of Hernia.

ESQUIROL, Dr. observations on Insanity.

EURYPHON, observations on the mode of treatment of Syphilis, employed by Fernelius and Palmarius.

FARADAY, Mr. observations on the Sounds produced by Flame in Tubes.

FERGUSON, Dr. observations on the Prevention of Typhous Fever.

FOURNIER, Dr. cases of Strangulated Hernia.

FRANCK, Dr. observations on Tetanus.

GEOGHAGAN, Mr. account of a case of Abscess in the Liver.

GIRDLESTONE, Dr. case of Exomphalos, with Stricture and Rupture of the Colon.

GOOD, Mr. observations on Inedia.

GRATTAN, Dr. observations on the Epidemic Fevers of Dublin.

HAMILTON, Dr. remarks on Lues Venerea.

HENNEN, Mr. observations on Military Surgery.

HUFELAND, Professor, case of Fracture.

HUMBOLDT, M. observations on the Milk of the Cow-tree.

HUME, Mr. observations on the Tests for Arsenic.

HUNT, Mr. case of Congenital Hernia.

HUTCHINSON, Mr. case of Nasal Operation.

JARDINE, Mr. an Essay to improve operative Surgery.

JAUZION, Dr. case of Nymphomania.

JOHNSON, Dr. case of Disease of the Brain.

KINGLAKE, Dr. observations on excessive Blood-letting in Maniacal Affection, and on Hepatic Congestion.

LALLEMAND, Dr. Pathological Observations.

LAURENT, M. observations on Ingurgitation.

LESLIE, Dr. observations on Tic Douloureux.

**Littleton**, Mr. observations on Pertussis; on Vision; and on Specific Gravity.
**Maclean**, Dr. cases of Hysteria, accompanied with Trismus; and of Calculus found in the Kidney of a Bitch.
**Mac Loghlin**, Dr. case of Ovarian Dropsy.
**Mahon**, Dr. observations on Amenorrhœa.
**Magendie**, Dr. researches into the nature of Gravel.
**Machell**, Mr. observations on the use of Enemas, with a description of an improved instrument for administering them.
**Meeker**, Dr. case of Preternatural Joint cured by Friction.
**Mills**, Dr. case of Hepatic Disease, &c.
**Newnham**, Mr. observations on Inversion of the Uterus, and the relation of a case of Extirpation of that Organ.
**O'Brien**, Dr. observations on the Epidemic Fever of Dublin; and relation of a case of Abscess of the Liver.
**Osbeck**, Mr. account of his mode of curing Syphilis by spare Diet.
**Parkinson**, Dr. Nosological Strictures; and observations on Scarlatina.
**Parkman**, Dr. observations on Insanity.
**Percival**, Dr. observations on the Epidemic Fever of Dublin.
**Percy**, Baron, observations on Ingurgitation.
**Pierson**, Dr. observations on the decarbonizing Function of the Lungs, &c.
**Pinel**, Dr. sketch of his Doctrine respecting Mental Alienation.
**Post**, Dr. cases of Aneurism.
**Prevost**, M. observations on the Colours of Bodies.
**Prideaux**, Mr. observations on the Tests for Arsenic.
**Purton**, Mr. observations on Uterine Hæmorrhage from Placental Presentation.
**Regnault**, Dr. observations on the use of Moxa in Hydrocephalus.
**Reid**, Dr. case of, with remarks on, Tetanus.
**Richerand**, Prof. account of the Excision of a portion of the Ribs and Pleura.
**Robiquet**, M. observations on the mode of preparing Prussic Acid.
**Roston**, Dr. observations on Aneurism of the Heart.
**Scott**, Mr. pathological observations on Fever.
**Serres**, Dr. observations on Paralysis.
**Smith**, Dr. observations on the use of Emetics in Spasmodic Diseases.
**Stoker**, Dr. relation of cases of Abdominal Tumors.
**Sym**, Mr. observations on Typhous Fever.
**Todd**, Mr. observations on Hernia.
**Travers**, Mr. Surgical Essays.
**Virey**, Dr. observations on the Medicinal Measures employed by Brute Animals.
**Wales**, Mr. observations on the profession of an Accoucheur.
**Williams**, Dr. observations on Dr. Charles Wilson's Specific for the Gout.
**Wilson**, Dr. **Andrew**, letters on Morbid Sympathies.

### PLATES.

Sketch of the Appearances of a Disease of the Humerus.
Representation of the Appearances of the Eruption in the Varioloid Disease prevalent in Derby.

Jas. Adlard and Sons, Printers,

23, Bartholomew Close.

